# Cuticle deposition ceases during strawberry fruit development

**DOI:** 10.1186/s12870-024-05327-7

**Published:** 2024-06-29

**Authors:** Jannis Straube, Grecia Hurtado, Viktoria Zeisler-Diehl, Lukas Schreiber, Moritz Knoche

**Affiliations:** 1 Institute of Horticultural Production Systems, Fruit Science Section, Leibniz University Hannover, Herrenhäuser Straße 2, Hannover, 30419 Germany; 2 Department of Ecophysiology, Institute of Cellular and Molecular Botany (IZMB), University of Bonn, Kirschallee 1, Bonn, 53115 Germany

**Keywords:** Cuticle, Cutin, Wax, Strain, *Fragaria* x *ananassa* Duch

## Abstract

**Background:**

Ideally, the barrier properties of a fruit’s cuticle persist throughout its development. This presents a challenge for strawberry fruit, with their rapid development and thin cuticles. The objective was to establish the developmental time course of cuticle deposition in strawberry fruit.

**Results:**

Fruit mass and surface area increase rapidly, with peak growth rate coinciding with the onset of ripening. On a whole-fruit basis, the masses of cutin and wax increase but on a unit surface-area basis, they decrease. The decrease is associated with marked increases in elastic strain.

The expressions of cuticle-associated genes involved in transcriptional regulation (*FaSHN1*, *FaSHN2*, *FaSHN3*), synthesis of cutin (*FaLACS2*, *FaGPAT3*) and wax (*FaCER1*, *FaKCS10*, *FaKCR1*), and those involved in transport of cutin monomers and wax constituents (*FaABCG11*, *FaABCG32*) decreased until maturity. The only exceptions were *FaLACS6* and *FaGPAT6* that are presumably involved in cutin synthesis, and *FaCER1* involved in wax synthesis. This result was consistent across five strawberry cultivars.

Strawberry cutin consists mainly of C16 and C18 monomers, plus minor amounts of C19, C20, C22 and C24 monomers, *ω*-hydroxy acids, dihydroxy acids, epoxy acids, primary alcohols, carboxylic acids and dicarboxylic acids. The most abundant monomer is 10,16-dihydroxyhexadecanoic acid. Waxes comprise mainly long-chain fatty acids C29 to C46, with smaller amounts of C16 to C28. Wax constituents are carboxylic acids, primary alcohols, alkanes, aldehydes, sterols and esters.

**Conclusion:**

The downregulation of cuticle deposition during development accounts for the marked cuticular strain, for the associated microcracking, and for their high susceptibility to the disorders of water soaking and cracking.

**Supplementary Information:**

The online version contains supplementary material available at 10.1186/s12870-024-05327-7.

## Background

The cuticular membrane (CM) is a lipophilic polymer that envelops the primary surfaces of all terrestrial plants. It covers the adaxial and abaxial surfaces of leaves and of most fruit. The CM functions as a barrier to restrict water loss [1] and to protect against UV light [2] and invasion by pathogens [3]. It also restricts non-stomatal gas exchange [4]. Fruit appearance is markedly enhanced by the cuticle, which gives it the desirable properties of smoothness and shininess—these consumers generally associate with freshness. To exhibit these properties, the fruit cuticle must remain intact throughout development. This poses a particular challenge for fruits as these mostly continue to expand until late in their development which (depending on species) corresponds to a period of several weeks to several months. Cuticular failure—microscopic cracking, or microcracking—during this period compromises the fruit skin’s barrier functions, resulting in uncontrolled water loss and shrivel, and these spoil its shiny appearance [5–7]. Cuticular failure is also associated with increased incidence of fruit rots resulting from pathogen invasion [8, 9]. Cuticular failure is usually followed by russeting, as the fruit’s dermal layers now differentiate to form a multicellular protective ‘periderm’ to regain control of water loss. However, the non-shiny, red-brown, rough-texture of the new russeted (secondary) fruit surface is usually considered by consumers to be an undesirable feature. Hence, for the grower, the economic consequences of cuticular failure are severe.

A plant cuticle comprises a polymeric cutin matrix, impregnated with intracuticular waxes and overlaid by epicuticular waxes. Cuticles also contain small quantities of polysaccharides [4, 10]. Cutin monomers are composed primarily of esterified hydroxylated or epoxy-hydroxylated fatty acids, specifically C16 and C18 [11–13]. These waxes consist mostly of very long-chain fatty acids (VLCFAs) ranging from C20 to C40, and including alcohols, alkanes, aldehydes, esters, ketones, flavonoids, sterols and triterpenes [4, 14].

Fruit crop species differ in their patterns of cuticular deposition. For example, apple fruits typically deposit CM materials throughout development, resulting in a progressive thickening of their CMs through to maturity. Deposition generally occurs on the inner (cell wall) surface of the cuticle. As a result, the outer (older) layers of the cuticle are markedly strained whereas the inner, more recently deposited (so younger) layers are less strained [15, 16]. The effect of this pattern of CM deposition, is that the more-recently deposited cutin on the inner side, ‘fixes’ the elastic strain in the older, outer layers. This limits both the formation and the anticlinal propagation of any cuticular microcracks.

In contrast, in stone fruit, e.g., sweet cherries and European plum, CM deposition ceases early during fruit development [17, 18]. In consequence, their cuticles are much thinner and they become markedly strained as the fruit expands during growth [19]. Strawberries are false fruits, pseudocarps, and are of unusual architecture. The strawberry 'fruit’ comprises a swollen receptacle tissue (not a swollen pericarp tissue, as in a true fruit). The true fruit of a strawberry are the pips (achenes) embedded in the receptacle surface. Strawberries also differ from the other fruit crops in that they: (1) develop over a very short time period and, hence, their skins are subject to very high rates of strain; (2) have the thinnest cuticles [20] of all 'fruit’ and (3) they suffer badly from surface disorders such as water-soaking and fruit-cracking, which are related to the formation of microscopic cracks in their cuticle [21, 22].

Little is known about CM deposition in strawberry fruit. A better understanding of this process will help in the development of strategies to reduce their susceptibility to surface disorders and thus improve fruit quality. These strategies should have relevance to strawberry breeders (e.g., selection criteria), to growers (e.g., crop management) and to postharvest handling (e.g., storage conditions).

The objectives of our study were: (1) to establish the developmental time course of cuticle deposition in strawberry; (2) to quantify any changes in cuticular strain that occur during fruit development and (3) to identify the molecular bases for any changes in cuticle deposition and relate these to compositional changes in the cutin and wax fractions of the developing cuticle.

## Results

**Fig. 1:**
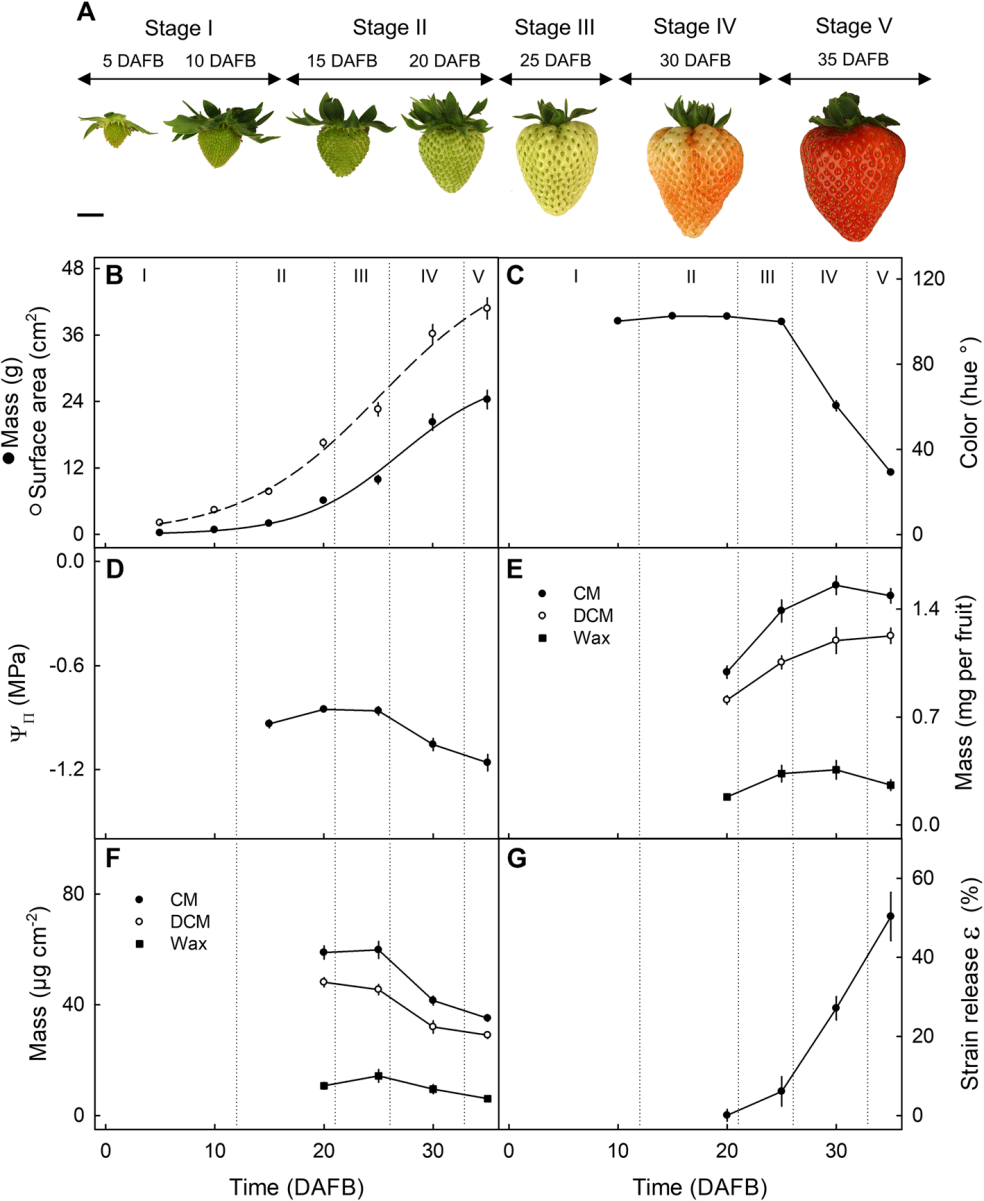
Developmental time course of increase in strawberry fruit mass and surface area, change in skin color, osmotic potential, deposition of the cuticular membrane (CM), the dewaxed cuticular membrane (DCM), and the wax on a whole-fruit basis and on a unit surface-area basis, and strain relaxation of the CM. **A** Photographs of typical fruit sampled at stage I (5 and 10 days after full bloom (DAFB)), stage II (15 and 20 DAFB), stage III (25 DAFB), stage IV (30 DAFB), and stage V (35 DAFB). **B** Fruit mass and surface area. The sigmoidal regression equations were: $Mass \left(g\right)=\frac{29.0}{\left(1+{exp}^{\left(\frac{-\left(Time \left(DAFB\right)-27.0\right)}{4.5}\right)}\right)}$ and: $Surface area \left({cm}^{2}\right)=\frac{50.1}{\left(1+{exp}^{\left(\frac{-\left(Time \left(DAFB\right)-25.2\right)}{6.3}\right)}\right)}$. **C** Skin color as indexed by the Hue angle. **D** Osmotic potential (ΨΠ). (E,F) CM, DCM, and wax mass on a whole-fruit basis (**E**) and a unit surface-area basis (**F**). **G** Strain release ε (%) of the CM on isolation. Data represent means ± SE. Scale bar in (A): 1 cm

Fruit mass and surface area increased rapidly with time following a lag phase up to about 15 days after full bloom (DAFB; Fig. 1A,B). The maximum increase in surface area occurred at about 25 DAFB as indexed by the first derivative of the sigmoidal regression model (Fig. 1B). This stage of development coincided with the onset of color change from green (hue° = 102.4 ± 0.14; Stage II) to white (hue° = 99.9 ± 0.36; Stage III) and to red at full maturity (hue° = 29.1 ± 1.26; Stage V) (Fig. 1C). The increase in growth rate also corresponded to a concurrent increase in soluble solids as indexed by a more negative osmotic potential (Ψπ) (Fig. 1D).

On a whole fruit basis, the masses of CM, dewaxed CM (DCM) and wax increased at a slowing rate up to about 30 DAFB, and then remained constant (Fig. 1E). However, on a unit surface area basis, the masses of CM, DCM and wax began to decrease at the onset of color change, suggesting that deposition did not keep pace with the increase in fruit surface area (Fig. 1F).

The decrease in deposition of CM, DCM and wax was accompanied by an increase in strain release (ε) after CM isolation (Fig. 1G). At maturity, the surface area of the isolated (relaxed) CM was only about half (50.3 ± 6.2%) that covered by it when previously stretched over the fruit surface (Fig. 1G).

**Fig. 2:**
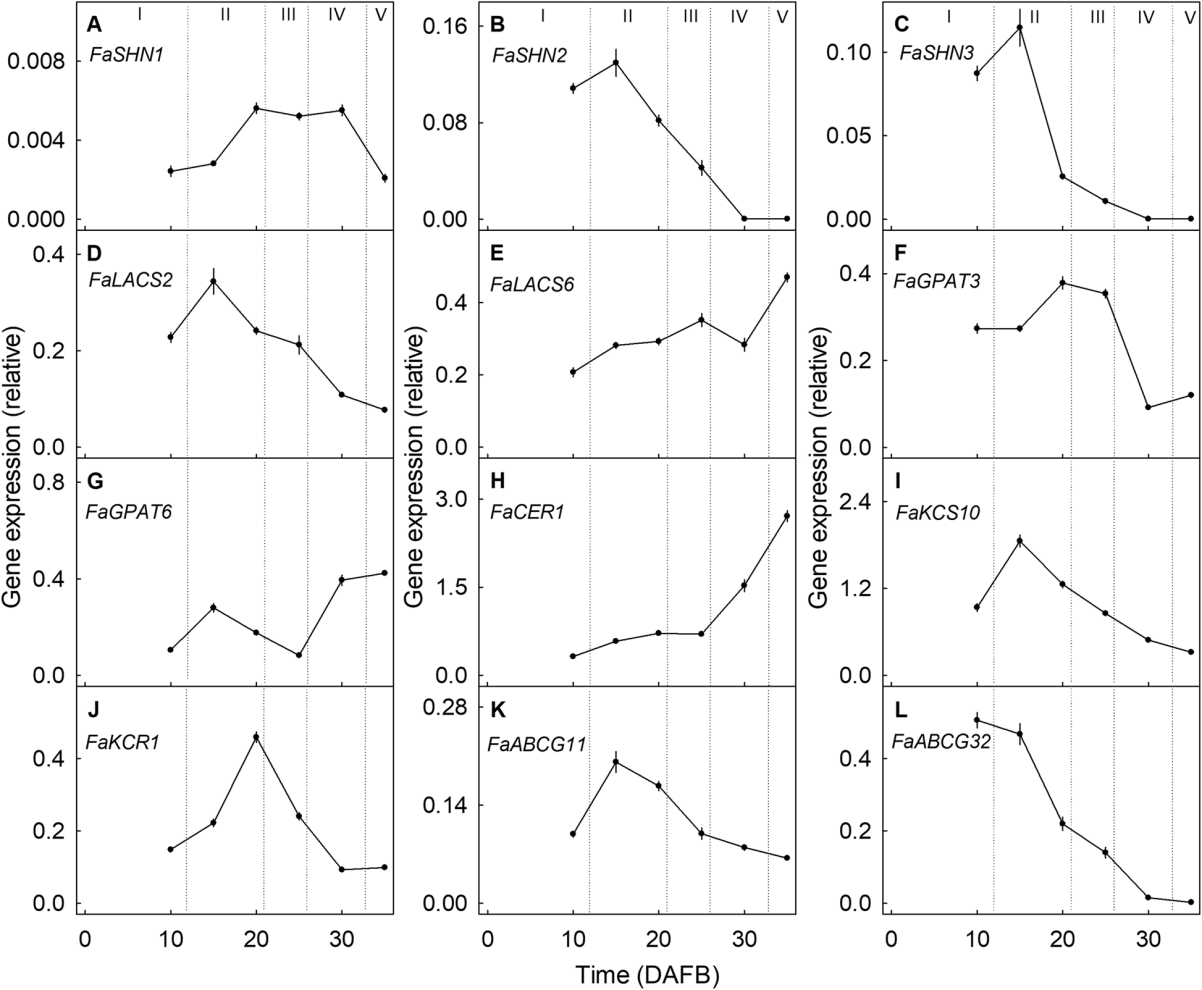
Developmental time course of expression of cuticle-associated genes in strawberry fruit. The genes investigated are involved in the transcriptional regulation *FaSHN1* (**A**), *FaSHN2* (**B**), and *FaSHN3* (**C**), the synthesis of cutin such as *FaLACS2* (**D**), *FaLACS6* (**E**), *FaGPAT3* (**F**), *FaGPAT6* (**G**) or the synthesis of wax such as *FaCER1* (**H**), *FaKCS10* (**I**), and *FaKCR1* (**J**), and the transport of cutin monomers and wax constituents *FaABCG11* (**K**) and *FaABCG32* (**L**). Stages of development are indicated in Roman numbers where stage I occurs at 10 days after full bloom (DAFB), stage II (15 and 20 DAFB), stage III (25 DAFB), stage IV (30 DAFB), and stage V (35 DAFB). Data represent means ± SE

The expressions of cuticle-associated genes, including those involved in transcriptional regulation (*FaSHN1*, *FaSHN2*, *FaSHN3*), the synthesis of cutin (*FaLACS2*, *FaGPAT3*) and wax (*FaCER1*, *FaKCS10*, *FaKCR1*), and those involved in the transport of cutin monomers and wax constituents (*FaABCG11*, *FaABCG32*) all decreased as the fruit matured, beginning as early as 15 DAFB (Fig. 2B-D, I, K-L) or 20 DAFB (Fig. 2A, F, J). The only exceptions were the two genes involved in cutin synthesis (*FaLACS6*, *FaGPAT6*) and one gene involved in wax synthesis (*FaCER1*), the expressions of these genes increased with time (Fig. 2E, G-H).

**Fig. 3:**
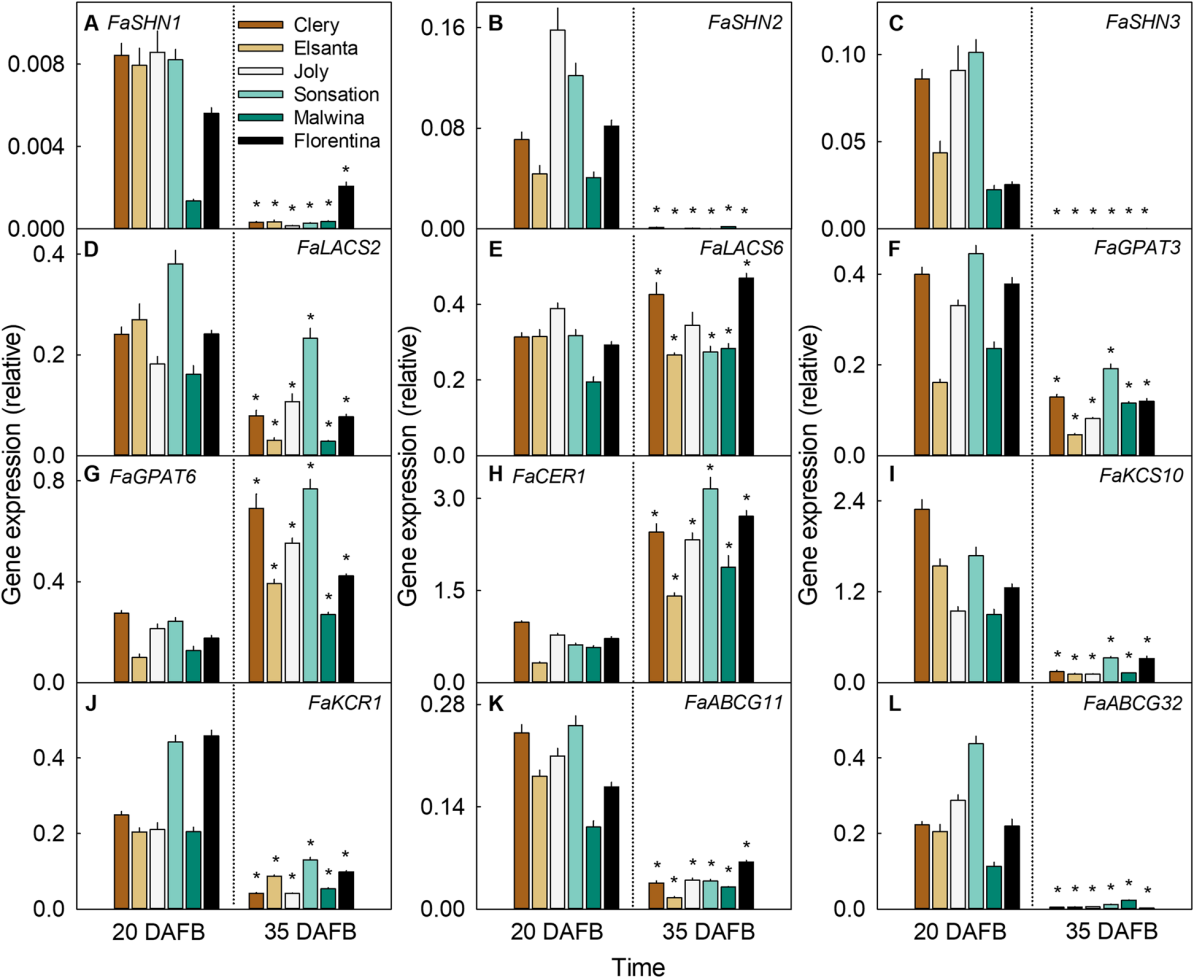
Gene expression of cuticle-associated genes involved in transcriptional regulation, synthesis and transport in six strawberry cultivars at 20 days after full bloom (DAFB, stage II) and 35 DAFB (stage V). The cultivars were 'Clery', 'Elsanta', 'Joly', 'Sonsation', 'Malwina' and 'Florentina' at 20 days after full bloom (DAFB; Stage II) and 35 DAFB (Stage V) for gene expression analysis. The data for 'Florentina' was redrawn from Fig. 1 for comparison. The genes investigated are involved in the transcriptional regulation *FaSHN1* (**A**), *FaSHN2* (**B**), and *FaSHN3* (**C**), the synthesis of cutin such as *FaLACS2* (**D**), *FaLACS6* (**E**), *FaGPAT3* (**F**), *FaGPAT6* (**G**) or the synthesis of wax such as *FaCER1* (**H**), *FaKCS10* (**I**), and *FaKCR1* (**J**), and the transport of cutin monomers and wax constituents *FaABCG11* (**K**) and *FaABCG32* (**L**). Data represent means ± SE. The asterisk '*' indicates significant differences between 20 and 35 DAFB within each cultivar at *p* ≤ 0.05 (Student's t-test)

The downregulation of *FaSHN1*, *FaSHN2*, *FaSHN3*, *FaLACS2*, *FaGPAT3*, *FaKCS10*, *FaKCR1*, *FaABCG11* and *FaABCG32* during fruit development was not unique to the cultivar 'Florentina', but occurred in all five cultivars investigated (Fig. 3A-D, F, I-L). Also consistent was the upregulation of *FaGPAT6* and *FaCER1* at maturity (Fig. 3G-H). The *FaLACS6* gene was upregulated in all cultivars except 'Joly', where expression remained constant (Fig. 3E).

**Fig. 4:**
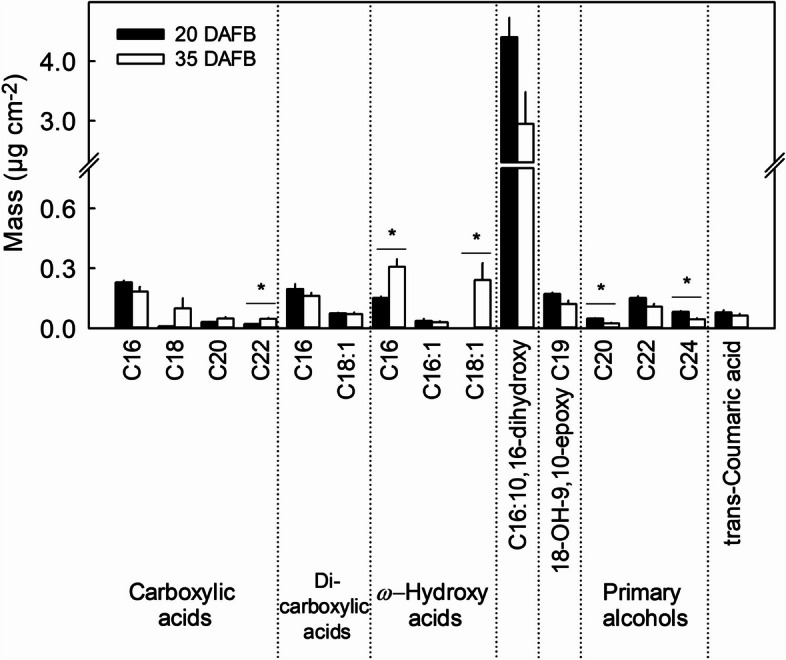
Cutin composition of skins excised from developing strawberry fruit. The composition of cutin was analyzed at 20 days after full bloom (DAFB, stage II) and at maturity 35 DAFB (stage V). Data represent means ± SE. The asterisk '*' indicates significant differences between 20 and 35 DAFB at *p* ≤ 0.05 (Student's t-test)

The strawberry cutin consisted primarily of C16 and C18 monomers, plus minor amounts of C19, C20, C22 and C24 monomers. It comprised various fatty acids, including *ω*-hydroxy acids, dihydroxy acids, epoxy acids, primary alcohols, carboxylic acids and dicarboxylic acids (Fig. 4). The most abundant cutin monomer in these developing strawberry fruit was 10,16-dihydroxyhexadecanoic acid. Between 20 DAFB and 35 DAFB, the amounts of these cutin monomers per unit area decreased by -20.8%. This decrease resulted primarily from a reduction in dihydroxy acids (-33.0%) and, most notably, in a decrease in 10,16-dihydroxyhexadecanoic acid (-33.0%). As the fruit matured, there were increases in carboxylic acids (+22.8%) and in *ω*-hydroxy acids (+67.2%), particularly in docosanoic acid (+54.5%), C16-hydroxy acid (+50.4%) and C18:1-hydroxy acid (+100%) (Fig. 4).

**Fig. 5:**
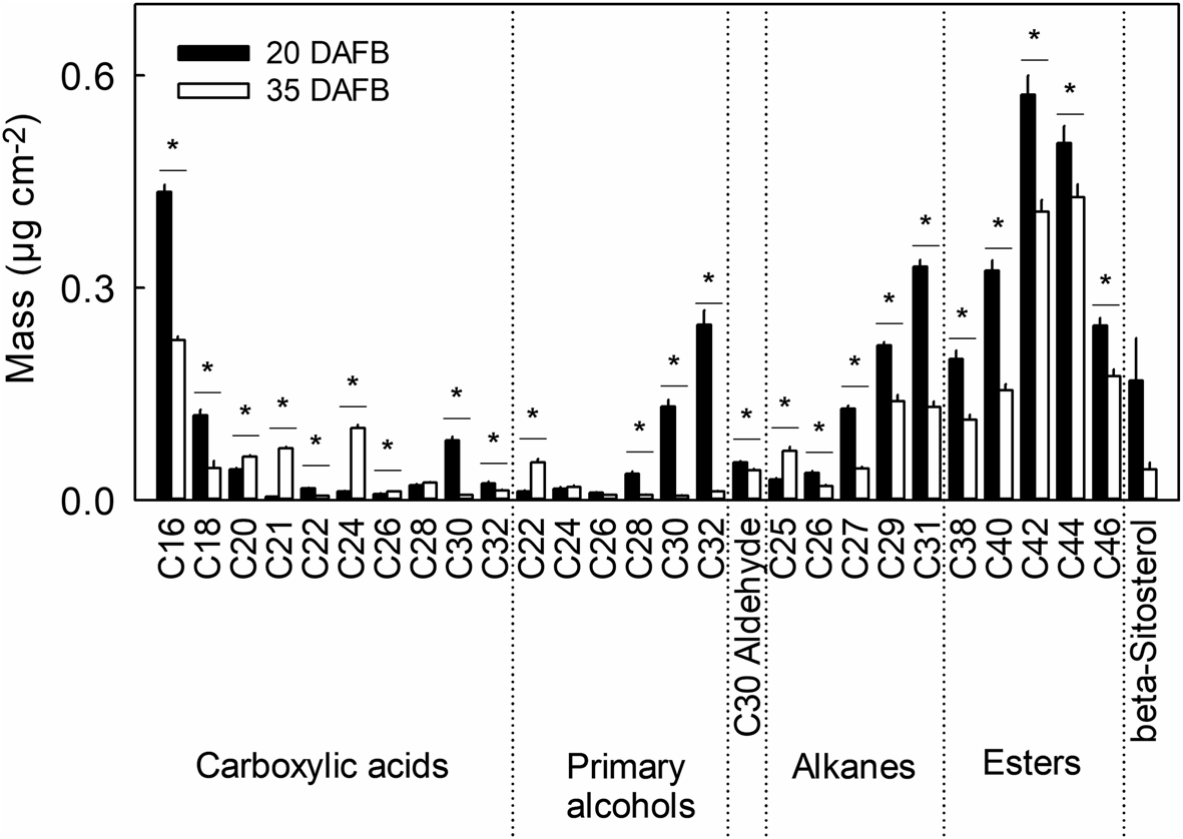
Wax composition of skins excised from developing strawberry fruit. The composition of wax was analyzed at 20 days after full bloom (DAFB, stage II) and at maturity 35 DAFB (stage V). Data represent means ± SE. The asterisk '*' indicates significant differences between 20 and 35 DAFB at *p* ≤ 0.05 (Student's t-test)

Wax constituents mainly comprised long-chain fatty acids ranging from C29 to C46, with lesser amounts of C16 to C28. Wax constituents were carboxylic acids, primary alcohols, alkanes, aldehydes, sterols and esters, the latter being most abundant (Fig. 5). The primary constituents of strawberry wax were C42 and C44 esters. Wax per unit area decreased by 39.6% between 20 and 35 DAFB. This decrease was accounted for primarily by decreases in the quantities of the primary alcohols (-68.1%) and alkanes (-57.6%), especially in 1-triacontanol (-95.5%) and 1-dotriacontanol (-94.9%). While most wax constituents decreased as the fruit matured, some minor constituents increased, including the carboxylic acids heneicosylic acid (+ 93.4%) and tetracosanoic acid (+ 87.8%) (Fig. 5).

## Discussion

Our discussion focusses on the following key findings.

1. Cuticle deposition does not keep pace with the increase in fruit surface area resulting in a thinning of the CM and the development of a marked strain.
2. Compared with other plant species, strawberry fruit have a typical cutin composition, but an unusual wax composition.
3. The decrease in cuticle deposition is accounted for by a decrease in the expressions of several cuticle related genes.

### The cessation of cuticle deposition in developing strawberry results in cuticle thinning and marked strain

In strawberry, cutin and wax deposition ceased at the onset of color change. The increase in fruit surface area that occurs after cessation of cuticle deposition, essentially distributes a constant amount of cuticle over an increasing surface area. This results in a thinning of the CM and a concurrent increase in strain. In these aspects strawberries are very similar to sweet cherries [19, 23, 24]. In contrast, the cuticle deposition pattern of tomatoes [25, 26], apples [19] and mangoes [27, 28] is different. In these latter fruit crops CM deposition continues throughout fruit development.

The decrease in cutin deposition in strawberry is primarily accounted for by a decline in the major cutin monomer, 10,16-dihydroxyhexadecanoic acid. Likewise, in sweet cherry major cutin constituents including the midchain oxygenated hydroxy acids, such as 9(10),16-dihydroxy-hexadecanoic acid and 9,10,18-trihydroxy-octadecanoic acid, decreased per unit surface area [24]. The decrease in wax mass per unit surface in strawberry is primarily caused by decreases in alkanes, primary alcohols, and esters. In contrast, the decrease in sweet cherries is attributed to decreases in the triterpenes, while the primary alcohols and alkanes remained constant [24].

The mismatch between CM deposition and fruit surface area expansion caused marked biaxial strain of the strawberry CM. Strained CMs are inherently more susceptible to microcrack formation, particularly when exposed to liquid water or to high water vapor concentrations [18, 29, 30]. Increased microcracking of strained cuticles during moisture exposure is related to a change in the rheological properties of the cuticle on hydration [31]. In apple, cutin deposition occurs throughput development on the inner (cell wall) side of the cuticle and wax is continuously embedded in the cutin polymer [15, 32]. Both processes fix the elastic strain previously developed by blocking strain relaxation. In this way, they convert elastic strain into plastic strain [16, 32]. However, strawberries lack comparable repair mechanisms due to the cessation of cutin and wax deposition. When the cuticle is strained beyond its limit of extension, microcracking occurs and this results in impaired barrier properties and thus increased susceptibilities to water soaking, to cracking and to fruit rots.

### Decreased cuticle deposition is the result of downregulation of cuticle-associated genes

We identified twelve genes potentially involved in cuticle formation in strawberries based on the functions of their orthologs. The expressions of nine of these were closely related to the rate of cutin and wax deposition both during the course of developmental and also in the cultivar comparison. These genes included three transcription factors, four genes involved in the synthesis of cutin monomers and wax constituents, and two genes involved in their transport.

Three transcriptional regulators from the APETELA2 (AP2)-domain TF superfamily, namely *FaSHN1*, *FaSHN2* and *FaSHN3*, were down-regulated at the same time as the fruit changed color. There was essentially no expression of these by fruit maturity. SHINE transcription factors are regulators of CM formation [33, 34]. They regulate CM formation in *Arabidopsis thaliana *and* Solanum lycopersicum*, *AtSHN1* governs wax and cutin synthesis in *A*. *thaliana* [35], *SlSHN2* [36] and *SlSHN3* [37] that in *S*. *lycopersicum*.

The downregulation of *FaLACS2* and *FaGPAT3* affected cutin synthesis. In *A*. *thaliana*, *AtLACS2* has been implicated in the esterification of fatty acids to coenzyme A, which is an early step in the synthesis of cutin monomers [38]. Similarly, the expression of *PaLACS2* in *Prunus avium* fruit is closely related to the rate of CM deposition [23]. Also, overexpression of *PaLACS2* in *A*. *thaliana* increases cutin deposition [39]. The gene *FaGPAT3* from strawberries shares a similarity with *AtGPAT9*, which participates in lipid synthesis in leaves and pollen grains [40, 41]. The absence of *FaGPAT3* in *Oryza sativa* affects the formation of the CM in anthers and of exines in pollen, resulting in decreased levels of cutin monomers and wax constituents [42].

The expressions of the wax synthesis genes *FaKCS10* and *FaKCR1* decreased towards maturity, as did wax deposition. In *A*. *thaliana*, *AtKCS10* synthesizes long-chain fatty lipids [43], while *AtKCR1* synthesizes very long-chain fatty acids that occur in cuticular wax [44].

The decrease in the expressions of the cutin and wax transporters *FaABCG11* and *FaABCG32* limited the availability of cutin and monomers for CM formation. In *A*. *thaliana*, AtABCG11 is involved in the transport of cutin monomers and wax constituents [45], while AtABCG32 is involved in the transport of only cutin monomers [46, 47].

The reason why the expressions of three genes, *FaLACS6* and *FaGPAT6*, and *FaCER1,* did not decrease in parallel with the decrease in cutin and wax deposition is not clear. It may be speculated that one or more of these genes is involved in the synthesis or deposition of some of those cutin and wax constituents that increased during development, i.e., perhaps of carboxylic (docosanoic acid) and *ω*-hydroxy acids (C16-hydroxy acid, C18:1-hydroxy acid) of the cutin fraction or the C25 alkanes of the wax fraction. However, definitive evidence is lacking. Studies by Fulda et al*.* [48] suggest that *AtLACS6* is involved in the *ß*-oxidation of fatty acids, but may not be directly involved in cutin and wax synthesis. *AtGPAT6* is involved in CM formation, particularly in the synthesis of 2-monoacylglycerols [49]. Deficiencies in *SlGPAT6* result in decreased cutin deposition and thinner CMs [50]. Similarly, *AtCER1* is involved in the synthesis of very-long-chain alkanes [51, 52]. Loss of *CsCER1* function in cucumber results in decreases in long-chain alkanes ranging from C25 to C33 in stems, leaves and fruits [53]. We would therefore have expected *FaGPAT6* and *FaCER1* to be downregulated in developing strawberry fruit but this was not the case.

### Strawberries have a typical composition of cutin, but an unusual composition of wax

Strawberries belong to the group of species that have a mixed cutin, comprised predominantly of C16 and C18 fatty acids [54]. So, strawberry cutin composition is generally similar to that of sweet cherry [24] and peach and pear [55]. Most of the monomers are midchain oxygenated hydroxyacids, notably 10,16-dihydroxyhexadecanoic acid. These hydroxyacids are also prevalent in the CMs of sweet cherries [24] and apples [56]. Interestingly, in sweet cherries, 10,16-dihydroxyhexadecanoic acid is the dominant monomer, while in apples, 9,10,18-trihydroxy-octadecanoic acid is the most abundant [56]. The latter is absent in strawberry cutin.

While the cutin composition of strawberry is similar to other *Rosaceae* fruit crops, the composition of the wax is somewhat unusual. First, strawberry wax lacks triterpenes. In many fruit crop species, triterpenes dominate the wax fraction. For example, ursolic acid dominates in apples [57], in sweet cherries [24] and in peaches [58], whereas amyrins (*α*-, *β*-type) dominate in pears [59]. Second, the secondary alcohol nonacosane-10-ol is absent in strawberries, but is a typical wax constituent of fruit waxes in the CMs of the *Rosaceae* family [60]. Third, characteristic of strawberry wax are esters ranging from C38 to C46. These are found in smaller quantities in apples [61] but not in sweet cherries [24], peaches [58] or pears [59] The alkanes found in strawberry wax are dominated by C27, C29 and C31 chain lengths, similar to those of *Gaultheria mucronata* [62]*, Nicandra physalodes*, *Physalis peruviana* and *Physalis ixocarpa* [63]. The decrease of wax mass per unit surface area in strawberry results primarily from decreases in alkanes, primary alcohols and esters caused by straining of the CM.

Comparing the cutin and wax yields obtained by gas chromatography with those obtained gravimetrically, revealed markedly lower yields by gas chromatography. This finding is not unique for the analyses of strawberry cutin and wax, but has also been reported for other fruit crop species [24]. The lower yields in gas chromatography may be attributed to one or several of the following factors: (1) Unesterified cutin may remain unchanged by methanolysis [64], (2) there are no response factors for quantifying secondary oxygenated acids such as those found in cutin [65], and (3) some depolymerization products may have restricted volatility under the GC conditions used [66]. Despite of these factors, the cutin composition identified in our study was similar to that reported by [54].

## Conclusion

In strawberries cutin and wax deposition cease at about the time of color change. The cessation of cuticle deposition is caused by the downregulation of nine putative CM genes involved variously in transcriptional regulation, cutin and wax synthesis and transport. These results for strawberry are consistent across the six cultivars we investigated. The fruit surface expansion that occurs after color change results in a thinning of the cuticle and a marked increase in strain and an increase in susceptibility to microcracking when the fruit surface is exposed to surface wetness or high humidity. This causal chain explains the high susceptibility of strawberries to disorders such as water soaking and cracking and to the high incidence of fruit rots.

## Materials and methods

### Plant materials

Strawberry fruit (*Fragaria* x *ananassa* Duch.) of 'Clery', 'Elsanta', 'Florentina', 'Joly', 'Malwina', and 'Sonsation' were cultivated in a growth chamber under controlled conditions of 20/16 °C day/night temperature, 80/60% day/night relative humidity (RH) and a 16 h photoperiod.

The developmental time courses of CM deposition, strain release, gene expression and change in cutin and wax composition were established in 'Florentina' strawberry. Fruit were sampled at 5, 10, 15, 20, 25, 30 and 35 DAFB. The developmental stages corresponded to stage I (5 and 10 DAFB), stage II (15 and 20 DAFB), stage III (25 DAFB), stage IV (30 DAFB) and stage V (35 DAFB) [67]. The cultivar comparisons of expression of cuticle-related genes were conducted using 'Clery', 'Elsanta', 'Joly', 'Malwina' and 'Sonsation'. These cultivars were selected because they represent major cultivars in todays strawberry production. Fruit were sampled at 20 and 35 DAFB corresponding to stages II and V. These stages represent the earliest, i.e., youngest, stage at which the analyses are technically possible (stage II) and the last stage when the strawberries are fully mature (stage V).

### Phenotypic observations

Fruit fresh mass was quantified gravimetrically. Individual flowers were labelled at full bloom. Ten representative fruits were sampled every five days until maturity and weighed. Representative digitally calibrated images (Canon EOS 550D, lens: EF-S 18–55 mm, Canon Inc., Ōta, Tokyo, Japan) were taken at each time point. The fruit’s [68] surface area was calculated from fruit mass using the empirical equation (Eq. 1).

1 $$A=5.0756*{\left(m\right)}^{0.6547}$$

Color was determined in the L*, a* and b* color space (CM-2600 d; Konica Minolta, Tokyo, Japan) and the hue angle calculated [69].

Juice was extracted from the fruit using a garlic press. The content of soluble solids (°Brix) was determined by refractometry (DR6200-T; A. Kruess Optronic, Hamburg, Germany). In a preliminary experiment the relationship between the osmotic potential of a fruit’s juice and its soluble solids content was determined. A total of 650 strawberry fruit of developmental stages ranging from stage I to stage V were sampled and the osmotic potentials (Ψ_π_) and soluble solids contents determined using water vapor pressure osmometry (VAPRO 5520 and 5600; Wescor, Logan, UT, USA) and refractometry (DR6200-T; A. Kruess Optronic, Hamburg, Germany). The following equation was established (Eq. 2) (Data S1). This equation allows prediction of the osmotic potential of a strawberry fruit from its soluble solids content:

2 $$\Psi \pi \left(MPa\right)=0.3292-0.0421xBrix-0.0088xBri{x}^{2}+0.0002xBri{x}^{3}$$

### Cuticular membrane isolation and strain analyses

Cuticular membranes were isolated enzymatically as described below [70]. An epidermal skin disc (ES) was first excised from the cheek of 'Florentina' fruit using a 4 mm diameter biopsy punch (Kai Europe, Solingen, Germany). Each ES had one achene in the center. Four ES per fruit were excised from a total of 50 fruit at each stage of development. The isolation of CM was limited to developmental the stages of 20 DAFB and beyond because of the limiting size of fruit younger than 20 DAFB.

For CM isolation, an ES was transferred to an enzyme solution containing 50 mM citric acid buffer (pH 4.0), pectinase (9%, v/v; Panzym Super E flüssig; Novozymes A/S, Krogshoejvej, Bagsvaerd, Denmark), cellulase (cellulase (0.5% v/v; Cellubrix L.; Novozymes A/S), and 30 mM NaN_3_. The ES was incubated at room temperature and the enzyme solution was refreshed periodically until all cellular debris had separated from the CM. For the final cleaning step the CMs were treated by ultrasonication at 35 kHz for 10 min (RK 510; Sonorex Super, Bandelin electronic, Berlin, Germany). Once complete separation was achieved, the CMs were thoroughly rinsed in deionized water. The achenes were removed by hand. The CM samples were dried for 48 h above dry silica gel at 22 °C, and subsequently weighed on a microbalance (M2P; Sartorius, Göttingen, Germany). The experiment was carried out with 10–12 replicates where each replicate comprised five CM discs.

Wax was extracted by incubating the CM discs in a 1:1 CHCl_3_/MeOH (v:v) solution. The DCM so obtained were dried above dry silica gel and weighed. The mass of the wax was determined by subtracting the mass of the DCM from that of the CM.

The apparent biaxial strain release (ε;%) resulting from CM isolation was quantified according to Lai et al. [19]. Briefly, a hydrated CM was transferred to a microscope slide, flattened using a coverslip. Digital calibrated images were prepared using a binocular microscope (MZ10F; Leica Microsystems, Wetzlar, Germany) equipped with a digital camera (DP73; Olympus; Tokio, Japan). The area of the flattened disc after isolation (A_CM_) was measured by image analysis (cellSens Dimension 1.18; Olympus Soft Imaging Solutions, Münster, Germany). The value of ε was calculated from Eq. 3, where A_CM_ represents the area of the isolated CM and A_0_ the area of the disc on the fruit before excision (i.e., the area of the biopsy punch).

To establish the effect of the achene cavity on the apparent biaxial strain release, a preliminary experiment was done using fruit with achene cavities ranging from very deep to shallow. The A_0_ value was assumed to represent the surface area of a truncated cone having a base diameter of 4 mm (equivalent to the diameter of the biopsy punch). Meanwhile A_CM_ was measured by making incisions in the CM disc and flattening the CM on the slide. The preliminary experiment revealed that accounting for the surface area of the achene cavity had no significant effect on the extent of strain release in the CM, calculated using the cross-sectional area of the biopsy punch as an estimate for A_0_ and the area of the CM without incisions as an estimate of A_CM_ (Data S2).

3 $$\mathrm\varepsilon({\%})=\frac{{\text{A}}_0-{\text{A}}_{CM}}{{\text{A}}_{CM}}\times100$$

### RNA Isolation

Fruit skin samples (40–60 mg per replicate) were excised from the region of the fruit that was of maximum diameter (‘cheek’) using a razor blade. Skin sections were immediately frozen in liquid nitrogen. The achenes were removed under liquid nitrogen prior to RNA extraction. The InviTrap Spin Plant RNA Mini Kit (STRATEC Molecular, Berlin, Germany) was used for RNA isolation according to the manufacturer's protocol. Residual genomic DNA was removed with DNase using the DNA-free™ Kit (Thermo Fisher Scientific, Waltham, MA, USA). The quantity and quality of RNA were assessed using a Nanodrop 2000c spectrophotometer (Thermo Fisher Scientific, Waltham, MA, USA) by measuring the absorbance at 230, 260 and 280 nm. RNA integrity was evaluated on a 1.5% agarose gel. cDNA synthesis was performed using the LunaScript® RT SuperMix Kit (New England Biolabs, Ipswich, MA, USA), incorporating 600 ng of total RNA in a 40 µL reaction volume following the manufacturer's protocol. Experiments were conducted using three replicates each comprised of six skin sections taken from six individual fruit per replicate.

### Gene expression analyses

Cuticle-associated genes were determined using blastP (Genome Data Base for *Rosaceae*. GDR [71]) from ortholog sequences of *Arabidopsis thaliana* as a query against the “*Fragaria* x *ananassa* Camarosa genome v1.0.a1 proteins” as the search target [72]. Primer3 software (Primer3, http://primer3.ut.ee/) was used to design gene specific primers (Table S1).

Gene expression was quantified by quantitative real-time PCR, employing the QuantStudio™ 6 Flex Real-Time PCR System (Applied Biosystems, Waltham, MA, USA). For each gene, three replicates were analyzed with two to three technical replicates. Gene expression data were normalized based on the reference genes *FaHISTH4* (AB197150.1) and *FaPIRUV4* (AF141016.2) [73]. The reactions were conducted with 1 µL of undiluted cDNA in an 8 µL volume of Luna® Universal qPCR Master Mix (New England Biolabs, Ipswich, MA, USA), following the manufacturer's recommendations. The final concentration of each specific primer was set at 200 nM. PCR conditions consisted of one cycle at 95 °C for 60 s, followed by 40 cycles at 95 °C for 15 s and 60 °C for 60 s. After the amplification, a melting curve analysis was performed (95 °C for 15 s, 60 °C for 60 s, with an incremental increase from 60 to 95 °C in 0.5 °C steps). Primer efficiency was assessed using a four-fold dilution series of a cDNA pool, covering five dilution points. All steps were done with the QuantStudio™ Real-Time PCR Software version 1.3 (Applied Biosystems, Waltham, MA, USA). Relative gene expression was determined according to Pfaffl [74] and as modified by Chen et al. [75].

### Identification and quantification of wax constituents

The CMs were isolated from 'Florentina' fruit sampled at 20 and 35 DAFB (equiv. to stage II and stage V). Wax was extracted from 1 to 4.5 mg samples of CM in 5 mL CHCl_3_ at room temperature on a horizontal rolling bench (CAT RM. 5–30 V, Staufen, Germany) overnight. To facilitate the subsequent quantification of individual wax constituents, an internal standard of tetracosane (100 µL of 10 mg tetracosane in 50 mL CHCl_3_) was added. The volume of CHCl_3_ was gradually reduced under a gentle stream of N_2_ at 60 °C. The DCMs were dried on Teflon discs for further analysis of cutin monomers.

Wax samples were derivatized by silylation, resulting in the corresponding trimethylsilyl ethers and esters. Next, 20 µL BSTFA (N, O-bis(trimethylsilyl)-trifluoroacetamid; Macherey–Nagel, Düren, Germany) and 20 µL pyridine (Sigma Aldrich; Deisenhofen, Germany) were added to each sample and incubated for 45 min at 70 °C. For quantification 1 µL of each derivatized sample was injected on-column into a gas chromatograph coupled to a flame ionization detector (GC-FID; CG-Hewlett Packard 5890 series H, Hewlett-Packard, Palo Alto, CA, USA, 307 column-type: 30 m DB-1 i.d. 0.32 mm, film 0.2 µm; J&W Scientific, Folsom, CA, USA). Wax constituents were identified by GC–MS (Quadrupole mass selective detector HP 5971, Hewlett-Packard, Palo Alto, CA, USA) with 1 µL injected on-column. Quantification of constituents was achieved using the internal standard, and molecule identification relied on the comparison of fragmentation patterns with literature data and an in-house data library. The number of replicates was five, where each replicate represented ten CM discs.

### Identification and quantification of cutin monomers

Following wax extraction, the DCMs were transesterified by incubation in 1 mL BF_3_/MeOH for 16 h at 70 °C. As an internal standard, 20 μg of dotriacontane (100 μL of 10 mg dotriacontane in 50 mL CHCl_3_) was added. The depolymerization of cutin was stopped by adding 2 mL of saturated NaHCO_3_ to the methanolysate. Cutin monomers were extracted with 3 × 2 mL CHCl_3_. The resulting CHCl_3_ phase was separated, rinsed with 1 mL HPLC-grade water, dried with Na_2_SO_4_, and concentrated under a stream of N_2_ at 60 °C. Subsequently, samples were derivatized according to the previously mentioned procedure. Quantification of monomers and constituents involved GC-FID, and identification was carried out using GC–MS, as detailed above. Fragmentation patterns were analyzed comparing them with both published data and our in-house library. Data were normalized relative to the internal standard and expressed per unit fruit surface area. The number of replicates was four (20 DAFB) or five (35 DAFB), where each replicate comprised a pool of ten CMs discs.

### Data analysis

Means between time points were compared using Student’s t-test at *p* ≤ 0.05 (R version 3.6.1; R Foundation for Statistical Computing, Vienna, Austria; [76]). The data in the figures are presented as means ± standard errors.

### Supplementary Information

Supplementary Material 1.

Supplementary Material 2.

Supplementary Material 3.

## Data Availability

All data supporting the findings of this study are available within the paper and its Supplementary Information.

## Abbreviations

BSTFA: N, O-bis(trimethylsilyl)-trifluoroacetamid
cDNA: Complementary DNA
CM: Cuticular membrane
DAFB: Days after full bloom
DCM: Dewaxed CM
ε: Strain release
ES: Epidermal skin disc
Eq.: Equation
_Ψπ_: Osmotic potential
SE: Standard error
VLCFAs: Very long-chain fatty acids

## References

[1] Riederer M, Schreiber L (2001). Protecting against water loss: analysis of the barrier properties of plant cuticles. J Exp Bot.

[2] Krauss P, Markstädter C, Riederer M (1997). Attenuation of UV radiation by plant cuticles from woody species. Plant Cell Environ.

[3] Serrano M, Coluccia F, Torres M, L'Haridon F, Métraux J-P (2014). The cuticle and plant defense to pathogens. Front Plant Sci.

[4] Yeats TH, Rose JKC (2013). The formation and function of plant cuticles. Plant Physiol.

[5] Knoche M, Grimm E, Winkler A, Alkio M, Lorenz J (2019). Characterizing neck shrivel in european plum. J Amer Soc Hort Sci.

[6] Sekse L (1995). Cuticular fracturing in fruits of sweet cherry (*Prunus avium* L.) resulting from changing soil water contents. J Hortic Sci..

[7] Sekse L (1995). Fruit cracking in sweet cherries (*Prunus avium* L.). Some physiological aspects-a mini review. Sci Hortic..

[8] Faust M, Shear C (1972). Russeting of apples, an interpretive review. HortScience.

[9] Winkler A, Athoo T, Knoche M (2022). Russeting of fruits: Etiology and management. Horticulturae.

[10] Domínguez E, Heredia-Guerrero JA, Heredia A (2011). The biophysical design of plant cuticles: an overview. New Phytol.

[11] Espelie KE, Dean BB, Kolattukudy PE (1979). Composition of lipid-derived polymers from different anatomical regions of several plant species. Plant Physiol.

[12] Kolattukudy PE (1981). Structure, biosynthesis, and biodegradation of cutin and suberin. Annu Rev Plant Physiol.

[13] Kolattukudy PE (2001). Polyesters in higher plants. Adv Biochem Eng Biotechnol.

[14] Jetter R, Kunst L, Samuel AL, Riederer M, Müller C (2006). Composition of plant cuticular waxes. Biology of the plant cuticle.

[15] Si Y, Khanal BP, Schlüter OK, Knoche M (2021). Direct evidence for a radial gradient in age of the apple fruit cuticle. Front Plant Sci.

[16] Khanal BP, Knoche M, Bußler S, Schlüter O (2014). Evidence for a radial strain gradient in apple fruit cuticles. Planta.

[17] Knoche M, Peschel S (2007). Deposition and strain of the cuticle of developing european plum fruit. J Am Soc Hortic Sci.

[18] Knoche M, Beyer M, Peschel S, Oparlakov B, Bukovac MJ (2004). Changes in strain and deposition of cuticle in developing sweet cherry fruit. Physiol Plant.

[19] Lai X, Khanal BP, Knoche M (2016). Mismatch between cuticle deposition and area expansion in fruit skins allows potentially catastrophic buildup of elastic strain. Planta.

[20] Hurtado G, Grimm E, Brüggenwirth M, Knoche M (2021). Strawberry fruit skins are far more permeable to osmotic water uptake than to transpirational water loss. PLoS ONE.

[21] Hurtado G, Knoche M (2023). Necked strawberries are especially susceptible to cracking. PeerJ.

[22] Hurtado G, Knoche M (2021). Water soaking disorder in strawberries: Triggers, factors, and mechanisms. Front Plant Sci.

[23] Alkio M, Jonas U, Sprink T, van Nocker S, Knoche M (2012). Identification of putative candidate genes involved in cuticle formation in *Prunus avium* (sweet cherry) fruit. Ann Bot.

[24] Peschel S, Franke R, Schreiber L, Knoche M (2007). Composition of the cuticle of developing sweet cherry fruit. Phytochemistry.

[25] Baker EA, Bukovac MJ, Hunt GM, Cutler DF, Alvin KL, Price CE (1982). Composition of tomato fruit cuticle as related to fruit growth and development. The plant cuticle, in: linnean society symposium series no. 10.

[26] Knoche M, Peschel S (2007). Gibberellins increase cuticle deposition in developing tomato fruit. Plant Growth Regul.

[27] Tafolla-Arellano JC, Zheng Y, Sun H, Jiao C, Ruiz-May E, Hernández-Oñate MA (2017). Transcriptome analysis of mango (*Mangifera indica* L.) fruit epidermal peel to identify putative cuticle-associated genes. Sci. Rep..

[28] Athoo TO, Khanal BP, Knoche M (2021). Low cuticle deposition rate in 'Apple' mango increases elastic strain, weakens the cuticle and increases russet. PLoS ONE.

[29] Knoche M, Peschel S (2006). Water on the surface aggravates microscopic cracking of the sweet cherry fruit cuticle. J Am Soc Hortic Sci.

[30] Hurtado G, Knoche M (2023). Microcracking of strawberry fruit cuticles: mechanism and factors. Sci Rep.

[31] Khanal BP, Knoche M (2017). Mechanical properties of cuticles and their primary determinants. J Exp Bot.

[32] Khanal BP, Grimm E, Finger S, Blume A, Knoche M (2013). Intracuticular wax fixes and restricts strain in leaf and fruit cuticles. New Phytol.

[33] Broun P, Poindexter P, Osborne E, Jiang CZ, Riechmann JL (2004). WIN1, a transcriptional activator of epidermal wax accumulation in Arabidopsis. Proc Natl Acad Sci USA.

[34] Aharoni A, Dixit S, Jetter R, Thoenes E, van Arkel G, Pereira A (2004). The SHINE clade of AP2 domain transcription factors activates wax biosynthesis, alters cuticle properties, and confers drought tolerance when overexpressed in Arabidopsis. Plant Cell.

[35] Kannangara R, Branigan C, Liu Y, Penfield T, Rao V, Mouille G (2007). The transcription factor WIN1/SHN1 regulates cutin biosynthesis in *Arabidopsis thaliana*. Plant Cell.

[36] Bres C, Petit J, Reynoud N, Brocard L, Marion D, Lahaye M (2022). The SlSHN2 transcription factor contributes to cuticle formation and epidermal patterning in tomato fruit. Mol Hortic.

[37] Shi JX, Adato A, Alkan N, He Y, Lashbrooke J, Matas AJ (2013). The tomato SlSHINE3 transcription factor regulates fruit cuticle formation and epidermal patterning. New Phytol.

[38] Schnurr J, Shockey J, Browse J (2004). The acyl-CoA synthetase encoded by LACS2 is essential for normal cuticle development in Arabidopsis. Plant Cell.

[39] Declercq M, Alkio M, Sprink T, Schreiber L, Knoche M (2014). Effect of sweet cherry genes *PaLACS2* and *PaATT1* on cuticle deposition, composition and permeability in Arabidopsis. Tree Genet Genomes.

[40] Shockey J, Regmi A, Cotton K, Adhikari N, Browse J, Bates PD (2016). Identification of Arabidopsis *GPAT9* (At5g60620) as an essential gene involved in triacylglycerol biosynthesis. Plant Physiol.

[41] Singer SD, Chen G, Mietkiewska E, Tomasi P, Jayawardhane K, Dyer JM, Weselake RJ (2016). Arabidopsis GPAT9 contributes to synthesis of intracellular glycerolipids but not surface lipids. J Exp Bot.

[42] Men X, Shi J, Liang W, Zhang Q, Lian G, Quan S (2017). Glycerol-3-Phosphate Acyltransferase 3 (*OsGPAT3*) is required for anther development and male fertility in rice. J Exp Bot.

[43] Pruitt RE, Vielle-Calzada JP, Ploense SE, Grossniklaus U, Lolle SJ (2000). *FIDDLEHEAD*, a gene required to suppress epidermal cell interactions in Arabidopsis, encodes a putative lipid biosynthetic enzyme. Proc Natl Acad Sci USA.

[44] Beaudoin F, Wu X, Li F, Haslam RP, Markham JE, Zheng H (2009). Functional characterization of the Arabidopsis beta-ketoacyl-coenzyme A reductase candidates of the fatty acid elongase. Plant Physiol.

[45] Bird D, Beisson F, Brigham A, Shin J, Greer S, Jetter R (2007). Characterization of Arabidopsis ABCG11/WBC11, an ATP binding cassette (ABC) transporter that is required for cuticular lipid secretion. Plant J.

[46] Bessire M, Borel S, Fabre G, Carraça L, Efremova N, Yephremov A (2011). A member of the PLEIOTROPIC DRUG RESISTANCE family of ATP binding cassette transporters is required for the formation of a functional cuticle in Arabidopsis. Plant Cell.

[47] Fabre G, Garroum I, Mazurek S, Daraspe J, Mucciolo A, Sankar M (2016). The ABCG transporter PEC1/ABCG32 is required for the formation of the developing leaf cuticle in Arabidopsis. New Phytol.

[48] Fulda M, Schnurr J, Abbadi A, Heinz E, Browse J (2004). Peroxisomal Acyl-CoA synthetase activity is essential for seedling development in *Arabidopsis thaliana*. Plant Cell.

[49] Yang W, Pollard M, Li-Beisson Y, Beisson F, Feig M, Ohlrogge J (2010). A distinct type of glycerol-3-phosphate acyltransferase with *sn*-2 preference and phosphatase activity producing 2-monoacylglycerol. Proc Natl Acad Sci USA.

[50] Petit J, Bres C, Mauxion J-P, Tai FWJ, Martin LBB, Fich EA (2016). The glycerol-3-phosphate acyltransferase GPAT6 from tomato plays a central role in fruit cutin biosynthesis. Plant Physiol.

[51] Bourdenx B, Bernard A, Domergue F, Pascal S, Léger A, Roby D (2011). Overexpression of Arabidopsis *ECERIFERUM1* promotes wax very-long-chain alkane biosynthesis and influences plant response to biotic and abiotic stresses. Plant Physiol.

[52] Aarts M, Keijzer CJ, Stiekema WJ, Pereira A (1995). Molecular charaterization of the *CER1* gene of Arabidopsis involved in epicuticular wax biosynthesis and pollen fertility. Plant Cell.

[53] Wang W, Zhang Y, Xu C, Ren J, Liu X, Black K (2015). Cucumber ECERIFERUM1 (*CsCER1*), which influences the cuticle properties and drought tolerance of cucumber, plays a key role in VLC alkanes biosynthesis. Plant Mol Biol.

[54] Järvinen R, Kaimainen M, Kallio H (2010). Cutin composition of selected northern berries and seeds. Food Chem.

[55] Walton TJ, Kolattukudy PE (1972). Determination of the structures of cutin monomers by a novel depolymerization procedure and combined gas chromatography and mass spectrometry. Biochem.

[56] Holloway PJ (1973). Cutins of *Malus pumila* fruits and leaves. Phytochemistry.

[57] Belding RD, Blankenship SM, Young E, Leidy RB (1998). Composition and variability of epicuticular waxes in apple cultivars. J Am Soc Hortic Sci.

[58] Belge B, Llovera M, Comabella E, Graell J, Lara I (2014). Fruit cuticle composition of a melting and a nonmelting peach cultivar. J Agric Food Chem.

[59] Heng W, Huang H, Li F, Hou Z, Zhu L (2017). Comparative analysis of the structure, suberin and wax composition and key gene expression in the epidermis of ‘Dangshansuli’ pear and its russet mutant. Acta Physiol Plant.

[60] Wollrab V (1969). Secondary alcohols and paraffins in the plant waxes of the family of *Rosaceae*. Phytochemistry.

[61] Straube J, Chen Y-H, Khanal BP, Shumbusho A, Zeisler-Diehl V, Suresh K (2020). Russeting in apple is initiated after exposure to moisture ends: Molecular and biochemical evidence. Plants.

[62] Klavins L, Klavins M (2020). Cuticular wax composition of wild and cultivated northern berries. Foods.

[63] de Souza AX, Riederer M, Leide J (2022). Multifunctional contribution of the inflated fruiting calyx: Implication for cuticular barrier profiles of the *Solanaceous* genera *Physalis*, *Alkekengi*, and *Nicandra*. Front Plant Sci.

[64] Nip M, Tegelaar EW, de Leeuw JW, Schenck PA, Holloway PJ (1986). A new non-saponifiable highly aliphatic and resistant biopolymer in plant cuticles – evidence from pyrolysis and 13C NMR analysis of present day and fossil plants. Sci Nat.

[65] Graca J, Pereira H (2000). Methanolysis of bark suberins: analysis of glycerol and acid monomers. Phytochem Anal.

[66] Graca J, Schreiber L, Rodrigues J, Pereira H (2002). Glycerol and glyceryl esters of *w*-hydroxyacids in cutins. Phytochemistry.

[67] Winkler A, Hurtado G, Knoche M (2021). Xylem, phloem and transpiration flows in developing strawberries. Sci Hortic.

[68] Hurtado G, Knoche M (2023). Detached, wetted strawberries take up substantial water in the calyx region. Sci Rep.

[69] McGuire RG (1992). Reporting of objective color measurements. HortScience.

[70] Orgell WH (1955). The isolation of plant cuticle with pectic enzymes. Plant Physiol.

[71] Jung S, Lee T, Cheng C-H, Buble K, Zheng P, Yu J (2019). 15 years of GDR: New data and functionality in the Genome Database for *Rosaceae*. Nucleic Acids Res.

[72] Edger PP, Poorten TJ, VanBuren R, Hardigan MA, Colle M, McKain MR (2019). Origin and evolution of the octoploid strawberry genome. Nat Genet.

[73] Galli V, Borowski JM, Perin EC, Da Messias RS, Labonde J, Pereira IdS (2015). Validation of reference genes for accurate normalization of gene expression for real time-quantitative PCR in strawberry fruits using different cultivars and osmotic stresses. Gene..

[74] Pfaffl MW (2001). A new mathematical model for relative quantification in real-time RT-PCR. Nucleic Acids Res.

[75] Chen Y-H, Khanal BP, Linde M, Debener T, Alkio M, Knoche M (2019). Expression of putative aquaporin genes in sweet cherry is higher in flesh than skin and most are downregulated during development. Sci Hortic.

[76] R Core Team. R: A language and environment for statistical computing. URL. 2019. https://www.R-project.org/.

