# Supplementary material for: Cuticle deposition ceases during strawberry fruit development
Source: BMC Plant Biol. 2024 Jun 29;24:623. doi: 10.1186/s12870-024-05327-7 (PMC11218262; doi:10.1186/s12870-024-05327-7)
Supplement: Supplementary file 3 — Supplementary Material 3. [file 12870_2024_5327_MOESM3_ESM.docx]

**Table S1. List of primers used in this study.**

|  |  | **Primer sequence (5'-3')** | | | | |  | | | | |  |
| --- | --- | --- | --- | --- | --- | --- | --- | --- | --- | --- | --- | --- |
| **Gene name** | **Accession** | **Forward Primer** | | **Reverse Primer** | | **PCR efficiency (%)** | | | **Reference** | |  |  |
|  |  |  | |  | |  | | |  | |  |  |
| **Cuticle related** |  |  | |  | |  | | |  | |  |  |
| *FaABCG11* | maker-Fvb6-3-augustus-gene-377.37 | GGCCGCAAAAGAAAGCTCTC | | TCTCCGACCAGCCCATCTTA | | 87.4 | | | This study | |  |  |
| *FaABCG32* | maker-Fvb1-4-augustus-gene-9.50 | CAAGACCCCGAGCAGTTCTT | | CCCTGCTTCCCACATGAACA | | 88.4 | | | This study | |  |  |
| *FaLACS2* | augustus_masked-Fvb2-1-processed-gene-38.5 | AGATGGGCTGTTGGAGTTGC | | GCGTTGACCAAGCATTCGAG | | 86.2 | | | This study | |  |  |
| *FaLACS6* | maker-Fvb4-2-snap-gene-117.32 | TTGCTCAATCAGGGCCGTAG | | CCTTTGGGGTGCCAGTTGTA | | 83.3 | | | This study | |  |  |
| *FaCER1* | maker-Fvb5-2-augustus-gene-214.30 | CATCCACTGCCTCAAGTCCA | | GGCCAAGTCCATGTTCGTCA | | 81.7 | | | This study | |  |  |
| *FaKCR1* | augustus_masked-Fvb4-1-processed-gene-138.3 | CCTTGGTGGGTTTTGGGTCT | | CTTGGCGGGTCTGAGGAAAT | | 86.8 | | | This study | |  |  |
| *FaKCS10* | maker-Fvb2-1-augustus-gene-240.39 | ACTATGCTGTGGTGGTGAGC | | TGCGGAAGAAACAGTTGGGA | | 84.4 | | | This study | |  |  |
| *FaGPAT3* | maker-Fvb2-2-augustus-gene-102.37 | GGAGCCGTGGAATTGGAATG | | TCCACCCGATTGTCAACACC | | 90.8 | | | This study | |  |  |
| *FaGPAT6* | maker-Fvb6-4-augustus-gene-83.27 | GGAGCCGTGGAATTGGAATG | | TCCACCCGATTGTCAACACC | | 85.7 | | | This study | |  |  |
| *FaSHN1* | augustus_masked-Fvb6-4-processed-gene-225.4 | TCTCCTTCCCTCACTTGCCT | | AGTCTTCTGTCCCAGCTCCA | | 96.2 | | | This study | |  |  |
| *FaSHN2* | maker-Fvb2-2-augustus-gene-27.57 | TCCAAGCTCAAGAAGTGTTGC | | CGGGTCTCTTCTGCCAAACT | | 84.6 | | | This study | |  |  |
| *FaSHN3* | maker-Fvb2-1-augustus-gene-192.49 | GAAGTTCAGAGGAGTCAGGCA | | TGTCTCAAATGTGCCTAGCCA | | 91.3 | | | This study | |  |  |
| **Reference genes** |  |  | |  | |  | | |  | |  |  |
| *FaHISTH4* | AB197150.1 | GTGGCGTCAAGCGTATCTCC | | TGTCCTTCCCTGCCTCTTGA | | 85.3 | | | (Galli *et al.*, 2015) | |  |  |
| *FaPIRUV* | AF141016.2 | AGGTGCGTTGCGAAGAGGA | | CTAAATCTGTGAATGCGAATGAGG | | 87.7 | | | (Galli *et al.*, 2015) | |  |  |
|  |  | |  | |  | | |  | |  | | |
